# Effects of Paper Mulberry (*Broussonetia papyrifera*) Leaf Extract on Growth Performance and Fecal Microflora of Weaned Piglets

**DOI:** 10.1155/2020/6508494

**Published:** 2020-11-17

**Authors:** Guoshun Chen, Shengzhang Shui, Mingjie Chai, Dong Wang, Yingyu Su, Hongbin Wu, Xiaodong Sui, Yulong Yin

**Affiliations:** ^1^College of Animal Science and Technology, Gansu Agricultural University, Lanzhou, 730070 Gansu, China; ^2^Gansu Aonong Feed Technology Co., Ltd., Wuwei, 733000 Gansu, China; ^3^The Institute of Subtropical Agriculture, The Chinese Academy of Sciences, Changsha, 410000 Hunan, China

## Abstract

The paper mulberry (*Broussonetia papyrifera*) leaf is rich in alkaloids and flavonoids, which has high medicinal and feeding value. We aimed to analyze the effects of *B. papyrifera* leaf extract on growth performance, antioxidant capacity, immune functions, and fecal microflora of weaned piglets. Thirty healthy, 28-day-old piglets were randomly assigned to three groups and fed with a basal diet supplemented with 0, 150, and 300 g/t *B. papyrifera* leaf extract for 42 days (control group, group I, and group II) separately. The result revealed that the final weight of piglets in group II was higher than the other groups, and the diarrhea rate in this group was 62.9% lower than in the control group. The feed conversion ratio in group I was significantly lower than the other two groups. Higher blood urine nitrogen concentration was noted in group II, higher glutathione peroxidase and catalase in group II, higher superoxide dismutase in the control group, and higher immune globulins (Ig) IgG, IgA, and IgM in group II. There was no significant difference in community richness and community diversity among the three groups of fecal samples. The relative level of *Roseburia* was higher in groups I and II, while *Lactobacillus* was higher in the control group. In conclusion, supplementation with *B. papyrifera* leaf extract at a certain dosage can increase growth performance and antioxidant capacity of weaned piglets, reduce the occurrence of diarrhea, enhance immune functions and disease resistance, and affect the composition of fecal microflora.

## 1. Introduction

The term feed additives generally refer to ingredients that are intentionally added to animal feed to improve palatability, promote growth, and increase decomposition of antinutritional components [1, 2]. Numerous chemical feed additives are used for animal feed. Unfortunately, most of them have been proven to be harmful to animals or may produce antibiotic residues [3]. In recent years, because of increasing awareness of antibiotic resistances and residues of antibiotics in animal products, the potential risks of such antibiotic residues and trends toward more natural products have led to regulations prohibiting the use of antibiotics as feed additives [4].

Plant extract-based feed additives, one of the important alternatives to conventional antibiotics, are attracting increasing interest in animal feed production and production animal farming. There are about 250,000 to 500,000 plant species on Earth; however, only a small fraction (1%–10%) is used for human nutrition or for animal feed production and other purposes [5]. Natural plant extracts are typically processed to isolate active ingredients which are then used for feed production. The original plant material may contain a plethora of different chemical compounds which may play a critical role in animal feed. The main role of plant extracts in animal nutrition is to promote appetence, improve digestion, increase lactation, and prevent or treat certain diseases [6, 7].

The paper mulberry (*Broussonetia papyrifera*) is a deciduous tree belonging to the mulberry family (Moraceae) [8]. In Chinese medical research, numerous beneficial effects of *B. papyrifera* leaves have been identified, including antibacterial and antitumor effects, reducing blood sugar, enhancing immunity, and delaying the aging; these leaves are also commonly used as supplements in farm animal feed [9]. Fresh leaves of *B. papyrifera* are difficult to digest; thus, leaves are typically fermented by microbial degradation to increase digestibility [10]. However, fresh and fermented leaves may cause problems associated with cellulose digestion; therefore, the added amount must not exceed a certain threshold, which limits its applicability as a feed additive. It has been reported that supplementation with 15% *B. papyrifera* silage in the diet could improve the body weight, dry matter intake, and feed conversion rate of cattle [11]. The good adding effect in cattle feed may be explained by the fact that cattle belong to ruminants and can digest cellulose well. Thus, it may be more effective to extract the active components from *B. papyrifera* as feed additives for pigs. The main active ingredients in *B. papyrifera* leaves are flavonoids and alkaloids [12, 13]. The flavonoids mainly include quercetin, isoglycyrrhizin, and carotenoids. Flavonoids are polyphenolic metabolites of various plants and occur commonly in fruit, grains, and vegetables [14]. At present, more than 10,000 natural flavonoids have been identified, and these compounds have become important components of various nutrients, cosmetics, and pharmaceuticals [15]. Their main functions include antioxidative, anti-inflammatory, and anticancer activities [16]. Alkaloids are typically basic nitrogen-containing compounds that occur in living organisms that are generally associated with crucial physiological activities [17]. In animal breeding, some related plant extract products have been used that can reduce the number of bacterial colonies in poultry and pigs, reduce intestinal fermentation and intestinal-related lymphatic system activity, and promote intestinal mucus production [18]. Thus, the objective of the present study was to investigate effects of *B. papyrifera* extract on growth performance and fecal microflora of weaned piglets to identify an optimized supplementation regime for rearing weaned piglets.

## 2. Material and Methods

### 2.1. Experimental Design and Study Animals

A total of 30 weaned piglets (Duroc × Landrace × Yorkshire; 7.18 ± 0.33kg body weight) were randomly assigned to three groups of 10 individuals, each. Piglets were weaned 28 days after birth and were then fed pellets. Piglets in the control group were fed a basal diet (Table 1), group I was fed the basal diet supplemented with 150 g/t *B. papyrifera* leaf extract (containing alkaloids and flavonoids), and group II was fed the basal diet supplemented with 300 g/t *B. papyrifera* leaf extract. Feeding regime and environmental conditions were otherwise consistent between groups. Each group was fed at 6:00 a.m., 8:30 a.m., 12:00 p.m., 2:30 p.m., and 5:00 p.m. every day, and water was provided *ad libitum*. The barn and the pens were cleaned and disinfected on a daily basis to prevent diseases.

A total of 8500 g fresh *B. papyrifera* leaves were ground to extract active ingredients. Ground leaves were placed in a water bath at approximately 50–60°C, and three consecutive extractions were performed using 75% ethanol. The ratio of 75% ethanol to *B. papyrifera* leaves was 30 : 1 (volume: mass). The 1500 g produced extracts were pooled and were diluted with 1200 mL 50–60°C water. This solution was then extracted using 7500 mL petroleum ether three times to remove fat-soluble compounds and pigments. Finally, the extract solution was concentrated under reduced pressure and dried in vacuum to obtain 300–380 g final extract.

### 2.2. Growth Performance and Measurement of Biochemical Indicators, Antioxidant Capacity, and Antibody Concentrations

The piglets' body weight was measured at age 28, 35, 42, 49, 63, and 70 days. Occurrences of diarrhea were recorded, and rates were calculated according to the following equation: diarrhearate = Σ(thenumberofpigletswithdiarrhea × daysonwhichdiarrheawasobserved)/(numberofpigletsintherespectivetreatment × daysoftheexperimentalperiod) × 100 [19].

Venous blood (20 mL) was collected at the age of 70 days. Heparin sodium was added to 10 mL of venous blood to separate plasma at 25°C for 30 min. An additional 10 mL of venous blood was centrifugated at 25°C for 30 min to separate serum. ELISA kits (Shanghai Jianglai Biological Technology Co., Ltd., Shanghai, China) were used to determine activity of alanine aminotransferase (ALT), alkaline phosphatase (ALP), total superoxide dismutase (SOD), glutathione peroxidase (GSH-Px), and catalase (CAT) and to determine the serum content of total protein (TP), albumin (ALB), globulin (GLB), glucose (GLU), malondialdehyde (MDA), and blood urine nitrogen (BUN). Serum concentrations of immune globulins (Ig) IgG, IgA, and IgM and total antioxidant capacity (T-AOC) were also determined using the ELISA kit.

### 2.3. 16S rDNA Sequencing and Analysis

At the age of 63 days, three healthy piglets were selected from each group, and their feces were collected in sterile containers that were then stored in liquid nitrogen. DNA was isolated from feces using a QIAamp DNA Stool Mini Kit (QIAGEN, Germany). Bacterial 16S rDNA amplicons of the variable region V4 were produced using specific PCR primers (515F-GTGNCAGCMGCCGCGGTAA and 806R-GGACTACNVGGGTWTCTAA).

Sequencing libraries were constructed by using a TruSeq DNA PCR-Free sample preparation kit (Illumina, San Diego, CA, USA) followed by quality evaluation using a Qubit 2.0 device. The libraries were sequenced on an Illumina HiSeq 2500 platform using a HiSeq Rapid SBS Kit v2 with 500 cycles. Raw tags were obtained by merging paired-end reads using the FLASH (v1.2.7, http://ccb.jhu.edu/software/FLASH/) software. Chimera sequences were removed to select effective tags from clean tags. Operational taxonomic units (OTUs) were produced by clustering tags using a similarity threshold level. Taxonomic annotations of OTUs were produced using the Silva software (http://www.arb-silva.de) and the UNITE database (http://unite.ut.ee/index.php). Richness and diversity analyses were conducted based on alpha diversity which was evaluated using the R software with the four indicators Chao1, Ace, Shannon, and Simpson. The QIIME software was used to test beta diversity and to compare similarity between different samples.

### 2.4. Statistical Analyses

Data are shown as the mean ± standarddeviation. Data were analyzed with a one-way ANOVA using SPSS 21.0 (SPSS Inc., Chicago, IL, USA). Duncan's multiple comparison test was also used. Differences between treatments were considered significant at *P* < 0.05.

## 3. Results

### 3.1. Growth Performance and Occurrences of Diarrhea

Body weight of piglets in the three groups is shown in Table 2. On days 35 (*P* = 0.843) and 63 (*P* = 0.354), no significant difference in body weight was observed between groups I and II. At the end of the experiment, the body weight of piglets in groups I and II was significantly higher than that of piglets in the control group (*P* = 0.006). Diarrhea rate was significantly less common in group II than in the control group and in group I (*P* = 0.003). Average daily feed intake (*P* = 0.035) and feed conversion ratio (*P* = 0.047) of piglets in groups I and II were significantly lower than those of piglets in the control group, and the average daily feed intake of piglets in group II was lowest, with 4.98 kg. The result indicated that piglets supplemented with 300 g/t *B. papyrifera* extract had a better growth performance.

### 3.2. Blood Metabolites, Antioxidant, and Immune Functions

Blood metabolites and immune parameters reflect health status of animals and the utilization of nutrients. No significant differences in TP, ALB, and ALP were observed between the three groups (*P* > 0.05; Table 3). Serum levels of ALT, GLU, and GLB did not differ significantly between the control group and group II (*P* > 0.05). Serum levels of ALT and GLU in group I were significantly lower than those in the control group, by 36.47% (*P* = 0.042) and 43.14% (*P* = 0.038), respectively. The mean BUN concentration in group II was 8.83 mmol/L, which was significantly higher than that in the control group and group I (*P* = 0.023). The supplementation treatment thus affected serum biochemical characteristics in weaned piglets, which may also influence growth performance and health.

SOD activity was significantly higher in the control group than in groups I and II (*P* = 0.038), and serum GSH-Px (*P* = 0.046) and CAT (*P* = 0.033) activities were significantly higher in group II than in the control group.

Dietary supplementation with *B. papyrifera* can significantly increase IgA concentrations in weaned piglets. IgA (*P* = 0.036), IgG (*P* = 0.024), and IgM (*P* = 0.029) concentrations were highest in group II, with 2313.67 *μ*g/mL, 9528.00 mg/L, and 1326 mg/L, respectively. The result indicated that the addition of *B. papyrifera* leaf extract had certain effects on blood metabolism, antioxidant, and immune functions of piglets.

### 3.3. Sequencing Data

**Figure 1:**
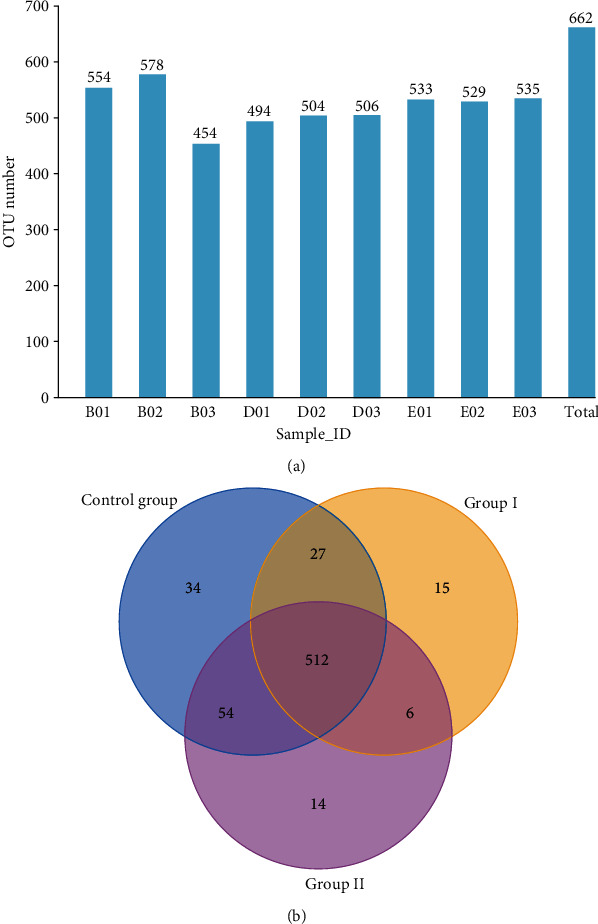
Numbers of OTUs in each sample (a) and a Venn diagram of the OTUs. B01 to E03 indicate the sample ID. Samples B01, B02, and B03 originated from the control group; samples D01, D02, and D03 from group I; and samples E01, E02, and E03 from group II.

A total of 720,482 reads were retrieved from nine samples. All reads were spliced, and 614,299 clean tags were obtained. A minimum of 65,482 clean tags were produced per sample (Supplementary 1). Sequences were grouped as OTUs of a similarity above 97%. Supplementary 2 shows the number of effective tags of the samples and the number of OTUs obtained by clustering each sample. Numbers of clean tags ranged from 44,597 to 52,501 after quality filtering. A total of 512 OTUs were shared by the three treatment groups, and 34, 15, and 14 OTUs were unique to the control group, group I, and group II, respectively (Figures 1(a) and 1(b)).

### 3.4. Bacterial Diversities and Community Compositions

**Figure 2:**
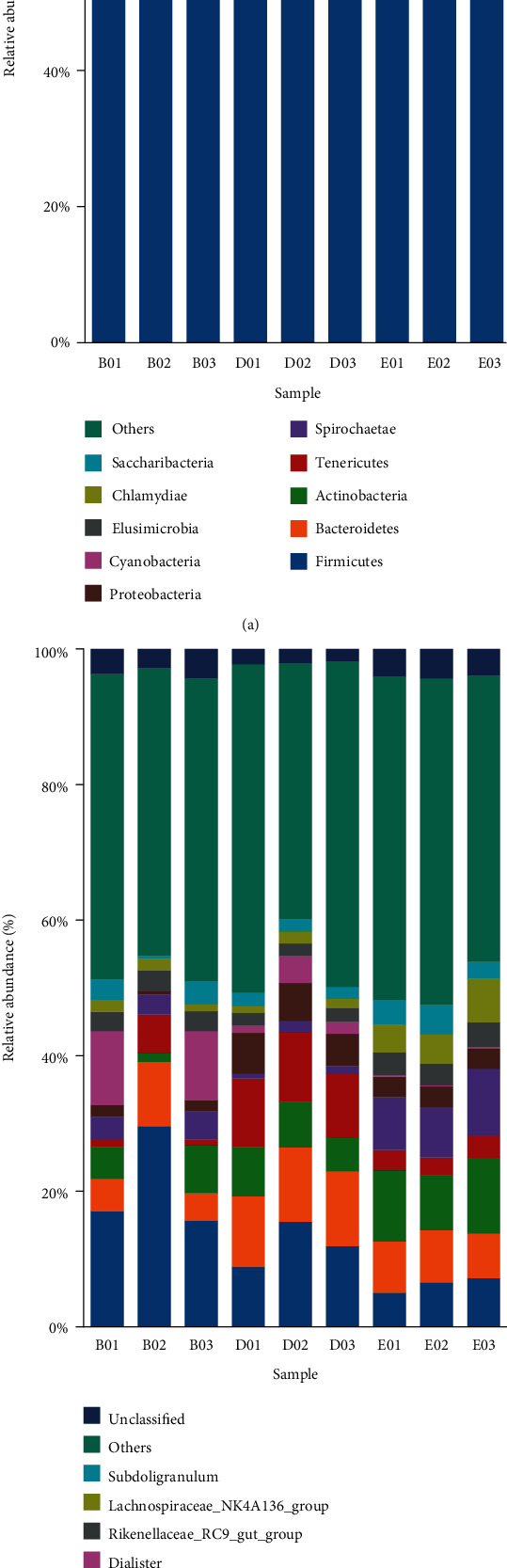
Top-10 maximum abundances of taxa at phylum (a) and genus levels (b).

The top-10 maximum abundance of taxa per each group at the phylum level is shown in Figure 2(a). *Firmicutes* occurred at the highest relative abundance in all nine samples, followed by *Bacteroidetes*, *Actinobacteria*, and *Tenericutes*. In groups I and II, the proportion of *Firmicutes* and *Proteobacteria* was lower and that of *Bacteroidetes* and *Fusobacteria* was significantly higher than those in the control group (*P* < 0.05). The proportion of *Tenericutes* in group II was higher than that in the control.

The top-10 maximum abundance of taxa at the genus level is shown in Figure 2(b). *Lactobacillus* showed the highest relative abundance in fecal samples. Compared with the control group, the relative abundance of *Lactobacillus* in groups I and II was decreased, and that of uncultured bacteria and of *Prevotella-9* showed an increasing trend. Levels of the *Prevotellaceae-NK3B31* group and *Roseburia* in group I were significantly higher than those in the control group. The relative abundance of *Megasphaera* in group II was significantly higher than that in the control group and in group I (*P* < 0.05). The result indicated that the proportion of bacteria in different groups was different.

### 3.5. Alpha and Beta Diversity Analyses

**Figure 3:**
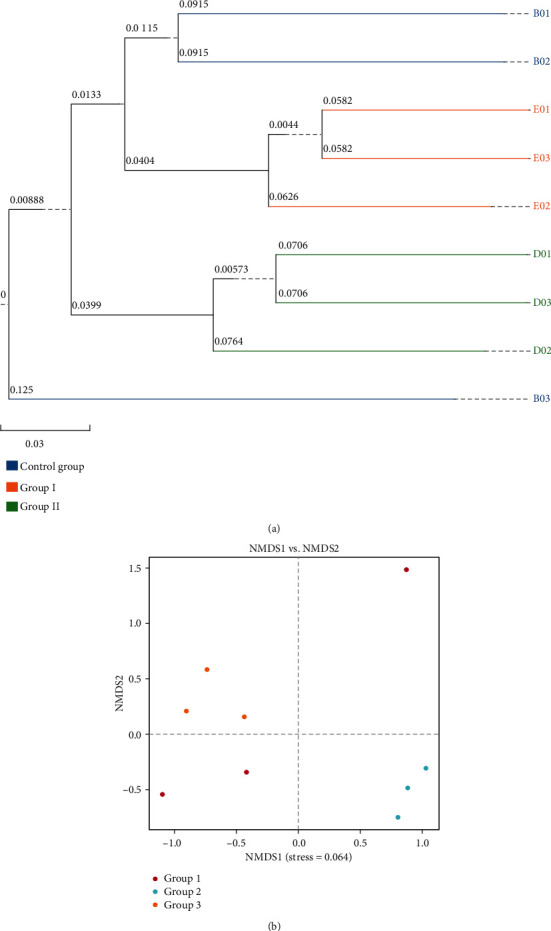
UPGMA cluster analysis (a) and nonmetric multidimensional scaling analysis (b) of piglet fecal samples.

Alpha diversity indices, including the observed OTUs, ACE, Chao1, Shannon, Simpson, and coverage for 9 samples, are shown in Table 4. In general, no significant difference in community richness, community diversity, and phylogenetic diversity was observed between the three experimental groups (*P* > 0.05). An unweighted pair group method with an arithmetic mean clustering dendrogram was produced from a fecal sample using a Bray-Curtis distance matrix. As shown in Figure 3(a), the similarity among fecal samples of the control group was higher than that between samples. Nonmetric multidimensional scaling was performed to assess differences between samples. The results showed that, apart from those of the control group, the samples of groups I and II were distinct, indicating that diet composition affected bacterial community composition in feces to some extent.

## 4. Discussion

*Broussonetia papyrifera* leaf extract contains a large amount of crude protein and is rich in various active compounds such as flavonoids and alkaloids. Since January 1, 2020, in addition to traditional Chinese medicines, the growth-promoting drug feed additives have been completely withdrawn (http://www.moa.gov.cn/govpublic/xmsyj/201909/t20190909_6327472.htm); thus, *B. papyrifera* leaf extract may be an invaluable substitute owing to its antibacterial and antiviral properties.

Numerous studies of the effects of plants on animal growth have been conducted, and a previous study showed that extracts of *Leucaena leucocephala* and *Salix babylonica* can positively affect production and fermentation of rumen gas, which improves nutrient utilization in lambs [20]. During the whole experiment, there was little difference in the average weight of the three groups, and there is no significant difference on the 63rd day. This result indicated that the supplementation of the extract of *B. papyrifera* leaves had little effect on the weight of piglets. For weaned piglets, diarrhea seriously affects the healthy development of piglets [21]. Although the addition of the extract had little effect on the weight of piglets, it could significantly reduce the diarrhea rate of piglets, which laid the foundation for the healthy development of piglets and the weight gain in the later stage. Lower feed conversion ratio is also an important performance of *B. papyrifera* additives to improve economic benefits.

Increased ALT concentrations may indicate liver damage [22]. In the present study, ALT concentrations in group I were significantly lower than those in the other two groups, indicating that supplementation with 150 g/t leaf extract may be beneficial to the liver of piglets, and the result of the 300 g/t treatment was consistent with that of the control group. Therefore, the supplementation treatment had no adverse effects on the piglets' livers. TP in plasma includes ALB and GLB [23]. Increased TP concentrations may cause immune disorders and liver dysfunction-related diseases [24]. However, TP concentrations did not differ between the three groups in the current study, indicating that the supplementation treatment did not adversely affect the piglets' livers. The plasma level of GLU is associated with digestion and the absorption rates of dietary starch, which indicates absorption, transport, and metabolism of sugar. GLU is predominantly important for oxidation and energy supply [25]. A previous study reported that addition of plant extracts can increase GLU levels [26], whereas a different study showed the opposite effect [27]. This may be because of differences in factors including the composition of the plant extract, the amount of supplementation, and the duration of the feeding experiment.

GSH-Px and SOD are important enzymes of the antioxidant system, and their activities are directly proportional to the body's ability to fight against free radicals [28]. MDA is a product of lipid peroxidation in cell membranes, and its concentrations can indirectly reflect the degree of oxidative tissue damage [29]. T-AOC indicates the overall level of enzymes and nonenzymatic antioxidants in the body [30]. CAT is a part of the peroxidation system in animal red blood cells and certain other tissues. Catalyzing the degradation of hydrogen peroxide to water and molecular oxygen is the main function of CAT, which prevents hydrogen peroxide from forming an iron chelate with molecular oxygen. The reaction under the action of the compound produces the toxic –OH molecule. The body's antioxidant capacity can be assessed using the blood antioxidant index [31]. In the present study, supplementation with *B. papyrifera* extract reduced MDA concentrations in weaned piglets, indicating that this extract inhibits lipid peroxidation of cell membranes, which are beneficial to the body's normal metabolism. In addition to the observed decrease in SOD activity, the activities of GSH-Px, T-AOC, and CAT in the serum of piglets in groups I and II increased. The activity of CAT and GSH-Px in group II was significantly higher than that in the control group. Supplementation with adequate doses of *B. papyrifera* extract may thus be beneficial to the antioxidant capacity of weaned piglets and promote their growth and development.

Serum Ig levels reflect the current immune status, and Igs play an important role in protecting piglets from invading pathogens which may cause intestinal diseases. In the current study, serum IgA, IgG, and IgM levels were significantly higher in group II than in the control group, and IgG and IgM levels were significantly higher in group II than in group I. A previous study showed that dietary supplementation with Yucca extract can significantly increase the TP content in laying hens [32]. We found that plant extracts can significantly increase serum IgA, IgG, and IgM levels in piglets and improve immunity in weaned piglets which is consistent with the results of a previous study [33]. Therefore, dietary supplementation with 300 g/t *B. papyrifera* extract can significantly improve antioxidant capacity and immune performance in weaned piglets. The level of serum immunoglobulin is only one aspect of immune function. In the future research, we need to do more experiments, such as splenocyte proliferation, natural killer cell activity, and T cell activity in both the spleen and peripheral blood, in order to obtain a more accurate conclusion of the effect of *B. papyrifera* extract on immune function of piglets.

Microbial community structures in piglet feces and treatment effects were analyzed, and 11 different genera were observed, predominantly belonging to the phyla *Firmicutes*, *Bacteroidetes*, *Actinobacteria*, *Tenericumes*, *Spirochaetae*, and *Elusimicrobia*. These 11 genera are important for degradation or crystallization and conversion of nitrogenous substances. *Lactobacillus* is a probiotic in animals, and it is important for healthy growth and development of the body [34]. Bacteria assigned as “uncultured bacterium” of the Bacteroidales family S24-7 were the predominant taxon in the fecal microflora at the genus level. These bacteria occur in the intestines. In the present study, the proportions of the phyla *Firmicutes* and *Proteobacteria* decreased, and those of *Bacteroidetes* and *Fusobacteria* significantly increased in groups I and II than those in the control group (*P* < 0.05). The proportion of *Tenericutes* in the fecal flora of group II was increased. At the genus level, the proportion of *Lactobacillus* in groups I and II decreased and that of “uncultured bacterium” and *Prevotella-9* increased compared with those in the control group. The abundance of Prevotellaceae-NK3B31 group and *Roseburia* was significantly higher in group I than in the control group and in group II (*P* < 0.05). *Roseburia* is a common genus of Clostridium cluster XIVa within Firmicutes that harbour prevalent butyrate producers [35]. Metabolism of dietary glycans is pivotal in shaping the human gut microbiota. *Roseburia intestinalis* as an abundant butyrate-producing Firmicutes plays a great role in the degradation of the major dietary fibre xylan [36]. The proportion of *Megasphaera* was significantly higher in group II than in the control group and in group I (*P* < 0.05). *Macroglobular* are Gram-negative cocci that are strictly anaerobic. They can ferment lactose and fruit acid in the gut. A study has reported that *Megasphaera* is significantly positively correlated with short-chain fatty acid (SCFA) production [37]. Among the SCFAs, propionate and butyrate are most often considered to benefit human health [38]. In the present study, diet composition altered bacterial community structures in piglet feces to some extent, and the proportion of different bacteria may affect the health of piglets. In conclusion, dietary supplementation with *B. papyrifera* leaf extract at an adequate dosage can significantly promote growth performance and antioxidant capacity in weaned piglets. Moreover, this treatment may effectively reduce the occurrence of diarrhea and improve immune functions and disease resistance.

## Figures and Tables

**Table 1:** Composition of the basal diets (%).

Item	Inclusion level
Maize	33.00
Soybean meal (46%)	10.00
Fish meal	3.00
Fermented soybean meal	5.00
Puffed soybean	6.00
Puffed maize	26.00
Whey powder	3.00
Salt	0.20
Calcium hydrogen phosphate	1.20
Stone powder	1.00
Suckling premix nutrient (1%) ^a^	1.00
Rapeseed oil	1.00
Wheat middlings	5.00
Wheat bran	3.40
Porcine compound enzyme	0.10
Flavouring agent	0.03
Sweetener agent	0.02
Montmorillonite	0.20
Acidifier	0.40
Threonine	0.10
Lysine	0.20
Methionine	0.10
Choline (VB450%)	0.05
Digestive energy	13.81
Crude protein	17.80
Calcium	0.65
Total phosphorus	0.54
Effective phosphorus	0.36
Sodium chloride	0.20

^a^Suckling premix nutrient (1%) content is equivalent to each kilogram of compound feed (not less than): Fe 75.0 mg; Zn 65.0 mg; Mn 18.0 mg; Cu 5.0 mg; Se 0.3 mg, I 0.14 mg; VA 3200 IU; VD 450 IU; VE 68.0 mg; thiamine 2.15 mg; riboflavin 2.80 mg; biotin 0.25 mg; folic acid 0.66 mg; niacin 32.00 mg; thbrthdrexvbdr 12.00 mg; VB_6_ 1.10 mg; VB_12_ 0.02 mg; choline chloride 450.00 mg; and antioxidants 30.00 mg.

**Table 2:** Performance of piglets on experimental diets.

Item	Control	Group I	Group II	*P* value
Initial weight (kg, 28 d weight)	7.25 ± 0.34	7.13 ± 0.30	7.16 ± 0.34	0.173
35 d weight (kg)	9.27 ± 0.63	9.04 ± 0.75	9.31 ± 0.34	0.843
42 d weight (kg)	12.03^A^ ± 0.73	11.14^B^ ± 1.76	12.12^A^ ± 0.34	0.004
49 d weight (kg)	15.74^A^ ± 0.34	15.30^B^ ± 0.75	15.62^A^ ± 0.34	0.002
56 d weight (kg)	19.35^a^ ± 0.80	19.14^b^ ± 0.99	19.59^a^ ± 0.35	0.023
63 d weight (kg)	23.94 ± 0.34	23.62 ± 0.62	23.82 ± 0.34	0.354
Final weight (kg, 70 d weight)	27.92^B^ ± 0.35	28.33^A^ ± 0.49	28.52^A^ ± 0.34	0.006
Average daily gain (kg/day)	0.49 ± 0.01	0.50 ± 0.01	0.51 ± 0.01	0.478
Diarrhea rate	10.00^C^ ± 0.45	7.70^B^ ± 0.51	3.71^A^ ± 0.87	0.003
Feed intake (kg/day)	6.50 ± 0.26^c^	5.60 ± 0.29^b^	4.98 ± 0.23^a^	0.035
Feed conversion ratio	13.27 ± 0.45^c^	11.2 ± 0.48^b^	9.76 ± 0.43^a^	0.047

Note: different lowercase letters in the same row represent significant difference (*P* < 0.05); different uppercase letters in the same row represent significant difference (*P* < 0.01); data with the same letter or no letter indicate that the difference was not significant (*P* > 0.05).

**Table 3:** Indicators of piglets on experimental diets.

Item	Control	Group I	Group II	*P* value
Plasma biochemical index				
ALT (U/L)	57.66^a^ ± 5.85	36.63^b^ ± 5.16	53.91^a^ ± 5.09	0.042
BUN (mmol/L)	5.67^b^ ± 0.86	6.32^b^ ± 0.63	8.83^a^ ± 0.92	0.023
GLU (mmol/L)	6.63^a^ ± 0.47	3.77^b^ ± 0.37	5.97^a^ ± 0.27	0.038
TP (g/L)	50.92 ± 3.60	47.49 ± 2.54	46.72 ± 3.48	0.764
GLB (g/L)	25.88^b^ ± 1.09	28.01^a^ ± 1.00	27.57^ab^ ± 0.43	0.027
ALB (g/L)	25.04 ± 4.00	19.45 ± 2.54	19.17 ± 3.66	0.875
ALP (U/L)	90.07 ± 22.61	113.13 ± 14.70	116.90 ± 20.97	0.956
Serum antioxidant index				
MAD (nmol/mL)	3.49 ± 0.32	3.26 ± 0.20	3.32 ± 0.30	0.624
GSH-Px (U/mL)	385.70^b^ ± 56.67	465.20^ab^ ± 26.90	528.23^a^ ± 62.18	0.046
T-AOC (U/mL)	346.83^ab^ ± 29.87	389.73^a^ ± 9.83	338.93^b^ ± 23.80	0.041
SOD (U/L)	342.33^a^ ± 18.00	265.03^b^ ± 39.80	272.60^b^ ± 13.84	0.038
CAT (U/L)	88.02^b^ ± 5.66	88.11^b^ ± 6.26	105.36^a^ ± 6.51	0.033
Serum immunoglobulin index				
IgA (*μ*g/mL)	947.33^b^ ± 172.88	1286.67^b^ ± 264.25	2313.67^a^ ± 183.36	0.036
IgG (mg/L)	7422.67^b^ ± 386.03	9337.00^a^ ± 248.78	9528.00^a^ ± 504.55	0.024
IgM (mg/L)	793.33^c^ ± 97.34	1048.33^b^ ± 63.61	1326.00^a^ ± 156.62	0.029

Note: different lowercase letters in the same row show significant difference (*P* < 0.05).

**Table 4:** Alpha diversity indices.

Samples	OTU	ACE	Chao1	Simpson	Shannon	Coverage
B01	554	578.2566	585.3636	0.0321	4.5479	0.999
B02	578	598.9752	600.5	0.0619	4.1859	0.9991
B03	454	480.2386	483.5625	0.0335	4.3823	0.9991
D01	494	522.3333	530.8333	0.0257	4.5134	0.9988
D02	504	542.2831	554.8333	0.0298	4.3311	0.9988
D03	506	535.0668	546.1818	0.0275	4.5085	0.9989
E01	533	557.7354	564.3333	0.0206	4.7058	0.999
E02	529	556.5541	571.0	0.019	4.7589	0.999
E03	535	567.5031	575.5263	0.0248	4.5865	0.9989

Note: the B01 to E03 represent that the sample IDs B01, B02, and B03 represent the samples from the control group. D01, D02, and D03 represent the samples from group I. E01, E02, and E03 represent the samples from group II.

## Data Availability

The data used to support the findings of this study are available from the corresponding author upon request.

## Supplementary Materials

Supplementary materials Supplementary 1. Sample sequencing data processing results. Supplementary 2. Number of effective sequences and number of OTUs in fecal samples.
