# Supplementary material for: Effects of Paper Mulberry (Broussonetia papyrifera) Leaf Extract on Growth Performance and Fecal Microflora of Weaned Piglets
Source: Biomed Res Int. 2020 Nov 17;2020:6508494. doi: 10.1155/2020/6508494 (PMC7700021; doi:10.1155/2020/6508494)
Supplement: Supplementary materials — Supplementary 1. Sample sequencing data processing results. Supplementary 2. Number of effective sequences and number of OTUs in fecal samples. [file 6508494.f1.doc]

**Supplementary Table 1. Sample sequencing data processing results**

| Groups | Sample ID | Number of paired-reads | Raw sequence | Number of clean tags | Average length of sequence (bp) | GC (%) | Q20 (%) | Q30 (%) | Number of effective sequences/ Number of paired-reads (%) |
| --- | --- | --- | --- | --- | --- | --- | --- | --- | --- |
| Control | B01 | 80,136 | 73,973 | 65,482 | 419 | 53.48 | 96.69 | 93.75 | 74.13 |
|  | B02 | 80,283 | 74,331 | 66,122 | 421 | 53.41 | 96.68 | 93.69 | 78.43 |
|  | B03 | 80,050 | 74,223 | 65,958 | 419 | 53.36 | 96.67 | 93.72 | 75.33 |
| Group I | D01 | 79,608 | 75,841 | 69,007 | 418 | 53.52 | 97.27 | 94.71 | 79.87 |
|  | D02 | 80,089 | 75,976 | 69,112 | 420 | 53.6 | 97.21 | 94.59 | 80.57 |
|  | D03 | 80,290 | 76,077 | 69,120 | 418 | 53.54 | 97.22 | 94.63 | 80.04 |
| Group II | E01 | 80,086 | 76,422 | 70,099 | 417 | 53.48 | 97.32 | 94.79 | 81.44 |
|  | E02 | 80,052 | 76,752 | 70,285 | 416 | 53.53 | 97.3 | 94.75 | 80.58 |
|  | E03 | 79,848 | 75,814 | 69,114 | 419 | 53.48 | 97.26 | 94.68 | 81.43 |

Note: The B01 to E03 represent that the sanmple ID. B01, B02, B03 represent the samples from the control group. D01, D02, D03 represent the samples from the group I. E01, E02, E03 represent the samples from group II.
